# Emergent Heavy-Fermion Physics in a Family of Topological Insulators *R*AsS (*R* = Y, La, and Sm)

**DOI:** 10.1021/jacs.5c21743

**Published:** 2026-03-10

**Authors:** Iñigo Robredo, Yuan Fang, Lei Chen, Nazar Zaremba, Yurii Prots, Mitja Krnel, Markus König, Mikel I. Iraola, Thomas Doert, Jeroen van den Brink, Claudia Felser, Qimiao Si, Eteri Svanidze, Maia G. Vergniory

**Affiliations:** † 87145 Luxembourg Institute of Science and Technology (LIST), 5 Avenue des Hauts-Fourneaux, Esch-sur-Alzette L-4362, Luxembourg; ‡ Department of Physics & Astronomy, Extreme Quantum Materials Alliance, Smalley-Curl Institute, 3990 Rice University, Houston, Texas 77005, United States; § Stony Brook University, Department of Physics & Astronomy, Stony Brook, New York 11794, United States; ∥ Max Planck Institute for Chemical Physics of Solids, Dresden 01187, Germany; ⊥ Institute for Theoretical Solid State Physics, IFW Dresden, Dresden 01069, Germany; # Technical University of Dresden, Dresden 01062, Germany; ¶ 226245 Donostia International Physics Center (DIPC), Donostia, San Sebastián 20018, Spain; ∇ Département de Physique et Institut Quantique, Université de Sherbrooke, Sherbrooke, QC J1K 2R1, Canada; ○ Regroupement Québécois sur les Matériaux de Pointe (RQMP), Montréal, QC H3T 3J7, Canada

## Abstract

Realizing topological phases in strongly correlated materials has become a major impetus in condensed matter physics. Although many compounds are now classified as topological insulators, *f*-electron systems provide an especially fertile platform for emergent heavy-fermion phenomena driven by the interplay of topology and many-body effects. In this study, we examine the crystalline topology of a new *R*AsS series (*R* = Y, La, Sm), revealing a structural variant from previous reports. We demonstrate that YAsS and SmAsS host hourglass fermions protected by glide symmetry. SmAsS notably exhibits a strong effective-mass enhancement, placing it alongside SmB_6_ and YbB_12_ as a material that exemplifies how the Kondo effects pin the correlated *f*-electron states near the Fermi energy and, consequently, renormalize the energy and mass scales of topological surface states without destroying their crystalline protection. This tunability establishes SmAsS as a bridge between weakly correlated topological materials and Kondo insulators. To capture these features, we construct a minimal model incorporating *f*-electron degrees of freedom, which reproduces the observed topological properties and predicts that the surface states survive in the correlated regime, albeit shifted in energy. Our work thus introduces a new family of correlated topological materials and forecasts the robustness of their surface states under Kondo correlations.

## Graphical abstract

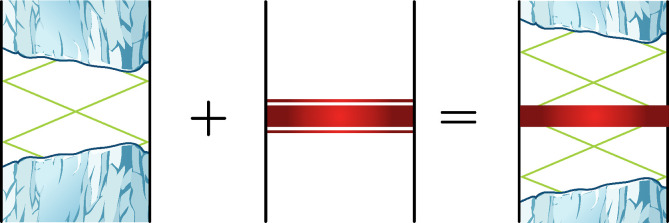

## Introduction

The discovery of topological materials has reshaped our understanding of quantum phases, offering potential breakthroughs in next-generation technologies, from fault-tolerant quantum computing to dissipationless electronic devices.^1–3^ However, despite the rapid progress in classifying and engineering weakly correlated topological systems,^4–9^ a crucial missing piece in this landscape is the realization of topological phases in strongly correlated materials. The combination of nontrivial band topology with electronic correlations is predicted to give rise to exotic quasiparticles, interaction-driven topological transitions, and even novel forms of superconductivity^10–12^ —yet experimentally confirmed examples remain scarce. Heavy-fermion systems, where flat bands from localized *f*-electrons hybridize with itinerant conduction states, provide a fertile ground for realizing such physics, as their many-body interactions can dynamically modify topological properties in ways that go beyond conventional band theory.^13–16^ Notable examples are Kondo systems, both Kondo insulators such as SmB_6_, YbB_12_ ^17–21^ and Weyl-Kondo semimetals^22–24^ such as realized in Ce_3_Pd_3_Bi_4_.^24,25^ However, heavy-fermion topological crystalline insulators (TCIs) whose nontrivial topology is enforced by crystalline symmetries remain rare.

In this work, we report the discovery of two new topological crystalline insulators with hourglass fermions, namely SmAsS and YAsS. Furthermore, the SmAsS compound is a heavy-fermion candidate and LaAsS is close to a topological transition. These materials are closely related to the ZrSiS aristotype family of square-net semimetals, which have recently been in the spotlight of the topological condensed matter community in regard to their topological properties.^26–29^ At the same time, we resolve a long-standing misclassification of the three compounds that were previously reported with inaccurate structures.^30^ Combining single crystal technique with high-resolution powder diffraction performed by means of a synchrotron source, we accurately establish their atomic arrangements, which differ significantly from prior reports. Using the formalism of topological quantum chemistry (TQC),^6,7^ we determine the topological invariants of these compounds and show that two of them host glide-symmetry protected hourglass fermions (Y/SmAsS) while the other is close to a topological transition (LaAsS). Using DFT calculations and effective tight-binding models (TB), and incorporating the correlated *f*-electrons, we are able to derive the simplest model that captures the fundamental topological properties of the three systems. Interestingly, this model serves as the physical realization of the layer construction used to explain similar topological properties in related systems, such as LaSbTe^31,32^ or ErAsS.^33^ The measurements of electrical resistivity of YAsS and SmAsS single crystals reveal semiconducting behavior for both, while a large value of the Sommerfeld coefficient γ = 160 mJ mol_Sm_^–1^ K^–2^ for SmAsS signals significant effective electron mass enhancement. Rather than a simple coexistence of *f*-electron bands and topological surface states, SmAsS reveals a regime in which correlation pins the strongly correlated single-particle excitations to the Fermi level and thus shifts and renormalizes the topological gap, offering a route to correlation-tuned topology.

## Results and Discussion

### Crystal Structure and Magnetic Behavior

The crystal structure models of rare earth arsenic sulfides *R*AsS reported previously as well as results of present publication show different kind of deformations starting from the tetragonal aristotype ZrSiS,^34^ realized e.g. for UAsS, space group *P*4/*nmm*, *a* = 3.879 Å, *c* = 8.168 Å.^35^ For this reason, we use in this manuscript nonstandard settings for the derivatives of the tetragonal aristotype to better illustrate the relationships between different crystal structure models and to discuss the particularities encountered in crystal structure determination.

Initially, a monoclinic structure was established for CeAsS (reported: space group *P*112_1_/*b*, *a* = 4.047 Å, *b* = 5.616 Å, *c* = 17.45 Å, γ = 135.85°, pseudotetragonal setting: space group *P*1121/*n*, *a* = 4.047 Å, *b* = 3.912 Å, *c* = 17.45 Å, γ = 90.26°)^36^ and later assumed for the whole *R*AsS series (with the exception of Eu and Yb), based on the powder X-ray diffraction data.^30^ Thus, transformation of the lattice reported for SmAsS (*a* = 3.93 Å, *b* = 5.48 Å, *c* = 17.00 Å, γ = 135.45°) into pseudotetragonal setting also resulted in the monoclinic angle close to 90°: *a* = 3.93 Å, *b* = 3.84 Å, *c* = 17.00 Å, γ = 90.37°. Later, the crystal structure of SmAsS was resolved as orthorhombic on the basis of single crystal data (space group *Pcmn*, *a* = 3.90 Å, *b* = 3.85 Å, *c* = 17.11 Å).^37^ Our re-examination of the crystal structure of the representatives of this series has shown that the laboratory equipment—either by powder diffraction or by single crystal experiments—is insufficient for a clear and precise identification of the respective atomic arrangement due to the non-negligible deformation of the unit cell. The task was made even more difficult by the tendency of the analyzed specimens to form twinned agglomerates. For this reason, high-resolution X-ray diffraction with synchrotron radiation (beamline ID22 at the ESRF) was needed in order to clearly identify the crystal structure. Based on this data, it was determined that the reported assignment of the monoclinic structure to the entire series is not accurate and a re-examination is required for each individual phase (see Figure 1).

**Figure 1:**
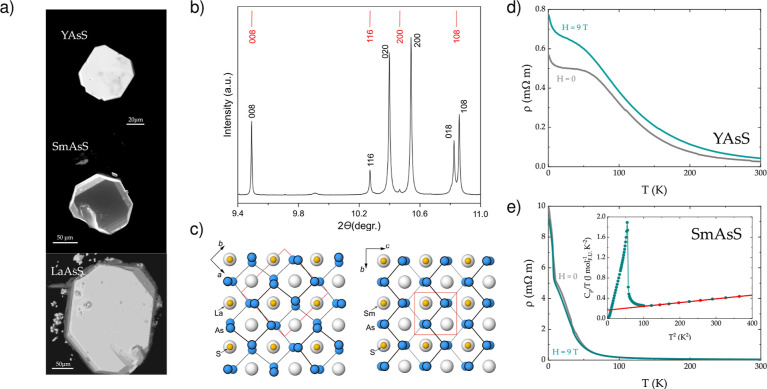
(a) Scanning electron micrographs of *R*AsS compounds. (b) Selected region diffraction pattern of SmAsS collected by using synchrotron radiation (λ = 0.35466 Å) at beamline ID22 at ESRF (for more details, see text). (c) Crystal structure of LaAsS (left) and SmAsS (right) projected along the largest (pseudotetragonal) translation period. The location of the zigzag and trapezoidal chains of As atoms differs by ^1^/_2_ of viewing direction, which is emphasized by thin and thick lines, respectively. Resistivity measurements for (d) YAsS and (e) SmAsS. Both compounds display semiconductor-like resistivity. For the SmAsS, entrance into the antiferromagnetic state is marked by a sharp anomaly around *T* = 7.5 K. The *C*_p_/*T* vs *T* ^2^ data are used to extract the value of the Sommerfeld coefficient γ = 160 mJ mol_Sm_^–1^ K^–2^ as the intercept of the *y*-axis (inset of panel (e)). This is reflecting the average effective electron mass across the Fermi surface.

Thus, the YAsS, SmAsS and LaAsS structures discussed here differ from those reported more than 40 years ago.^30^ YAsS (SmAsS) and LaAsS show different types of deformation, starting from the tetragonal aristotype ZrSiS. While an orthorhombic distortion along the original tetragonal axes *a* and *b* is observed for Y/Sm (space group *Pcmn*, *a* = 3.913 Å, *b* = 3.861 Å, *c* = 17.146 Å, nonstandard setting of the space group *Pnma*), a deformation along the diagonals of the tetragonal supercell (original tetragonal subcells of the ZrSiS type) is detected for the La phase (space group *Pnma*, *a* = 5.692 Å, *b* = 5.707 Å, *c* = 17.553 Å). As a result, the respective lattice parameters differ by *a* factor of $\sqrt{2}$ . The different deformation of the original tetragonal unit cell is due to the different arrangement of the As atoms in the structures. While the As atoms in the SmAsS-type structure are connected to form zigzag chains, the As chains in the LaAsS structure have a trapezoidal arrangement and are stretched in a 45° direction compared to the location of the As chains in the SmAsS structure, see Figure 1c. This kind of chain has been frequently referred to as a cis–trans chain.^38,39^ All these unit cell deformations are confirmed by the splitting of the corresponding reflections on the high-resolution X-ray powder diffractograms. In Figure 1b we show a selected region of the diffraction pattern of SmAsS collected by using synchrotron radiation (λ = 0.35466 Å). The indexes of the experimental pattern correspond to the nonstandard setting (*Pcmn*) of the space group *Pnma*, *a* = 3.913 Å, *b* = 3.861 Å, *c* = 17.146 Å. The bars in the upper part of Figure 1b correspond to the theoretically calculated positions of the tetragonal idealized lattice with the lattice parameter *a* = 3.887 Å, averaged from the respective values *a* and *b* of the real structure. This figure clearly shows the presence of the split reflections, which is typical for the orthorhombic distortion of the unit cell, and the absence of the additional reflection splitting of the *hhl* series, which would be expected in the case of the monoclinic distortion. For reference, the 00*l* reflections remain unsplit in both cases. In the case of LaAsS (space group *Pmnb*, *a* = 5.692 Å, *b* = 5.707 Å, *c* = 17.553 Å) with the orthorhombic deformation along the *ab* diagonal, the reflections of the *hhl* series are split, while the *h*0*l* reflexes remain unsplit. The difference in the deformation of the tetragonal unit cell of the ZrSiS aristotype, which led to two reported structural models, can be illustrated using the group-subgroup relationship (for a schematic representation, see Bärnighausen tree, shown in Supporting Information Figure S1). Note that the refinements haven been done using the standard *Pnma* setting for the three structures. We show full structural details in Tables S1–S3 in Supporting Information.

We performed temperature- and field-dependent resistivity measurements of YAsS and SmAsS (see Figure 1d and e). While the bulk magnetic order of SmAsS is confirmed by both specific heat, magnetization (not shown) and resistivity analysis, the exact magnetic configuration is still being investigated. In the normal state of SmAsS, we find a large value of the Sommerfeld coefficient γ = 160 mJ mol_Sm_^–1^ K^–2^. This value, extracted from the linear fit in the normal state—which is traditionally used as a measure of effective electron mass^15,16,40^ —is significantly above the value of γ observed in normal metals (for example SmMg_2_Bi_3_ ^41^ with γ = 2 mJ mol_Sm_^–1^ K^–2^ or SmCu_2_ ^42^ with γ = 6 mJ mol_Sm_^–1^ K^–2^). Thus, this provides an experimental measure of the effective electron mass enhancement in SmAsS (See Supporting Information for further details). This is in a good agreement with having flat bands at the Fermi level from very localized *f*-electrons from Sm atoms as DFT calculations predict, which can lead to heavy-fermion physics.

### Electronic Structure of YAsS

In the following sections we will explain the electronic structure of each compound in depth. We start our study with YAsS. Space group *Pnma*, common to all studied compounds, is formed by 2 fold screw axes $\left\{C_{2x}|\frac{1}{2}\frac{1}{2}\frac{1}{2}\right\}$ , $\left\{C_{2y}|0\frac{1}{2}0\right\}$ and $\left\{C_{2z}|\frac{1}{2}0\frac{1}{2}\right\}$ , glides $\left\{m_{x}|\frac{1}{2}\frac{1}{2}\frac{1}{2}\right\}$ (g_100_) and $\left\{m_{z}|\frac{1}{2}0\frac{1}{2}\right\}$ , mirror $\left\{m_{y}|0\frac{1}{2}0\right\}$ and inversion symmetry at the origin {*I*|000}. Since they carry no localized magnetic moment, time-reversal symmetry (TRS) is also present. The crystal structure and relevant glide symmetry are shown in Figure 2a. In Figure 2b, we display the Brillouin zone (BZ) of space group *Pnma*, indicating the high-symmetry points and the location of the glide symmetry, which leaves the *k*_*x*_ = 0, π planes invariant. In Figure 2c we show the bulk electronic band dispersion. In blue continuous lines we show the calculation in the absence of SOC (NSOC) and in black dashed lines we show the calculation including SOC. The two band structures are relatively close to each other, with one difference: the NSOC calculation shows band crossings while the SOC calculation is gapped (see inset Figure 2c). In order to characterize the topological properties of the system, we compute the symmetry indicators (SIs) both in the presence and absence of SOC by using the Fu-Kane-like formula for the $Z_{4}$ indicator^43–45^

1 $$Z_{4}=\sum_{K\in TRIM}\frac{n_{K}^{-}-n_{K}^{+}}{2}\mathrm{mod}4$$

where *n*_K_^–^ (*n*_K_^+^) denote the multiplicity of irreducible representations (irreps) with −1 (+1) eigenvalue of inversion symmetry. We show in Table 1 the irreps of the last disconnected set of bands, up to the Fermi level, both with and without SOC. We only list the irreps at Γ and *U*, since they are the only high-symmetry *k*-points that can host a band inversion. We compute the symmetry indicator and get that $Z_{4}=2$ , both with and without SOC. In both cases, the nontrivial symmetry indicator stems from a band inversion at the Γ point, with 4 more states with inversion symmetry eigenvalue +1 than states with eigenvalue −1. Interestingly, while the SOC gaps the crossings of the NSOC case, it does not alter the symmetry indicator. This is different from usual topological insulators, where the band inversion is driven by SOC.^46^ This suggests that we can construct a minimal NSOC model for the system that describes the main topological properties.

**Figure 2:**
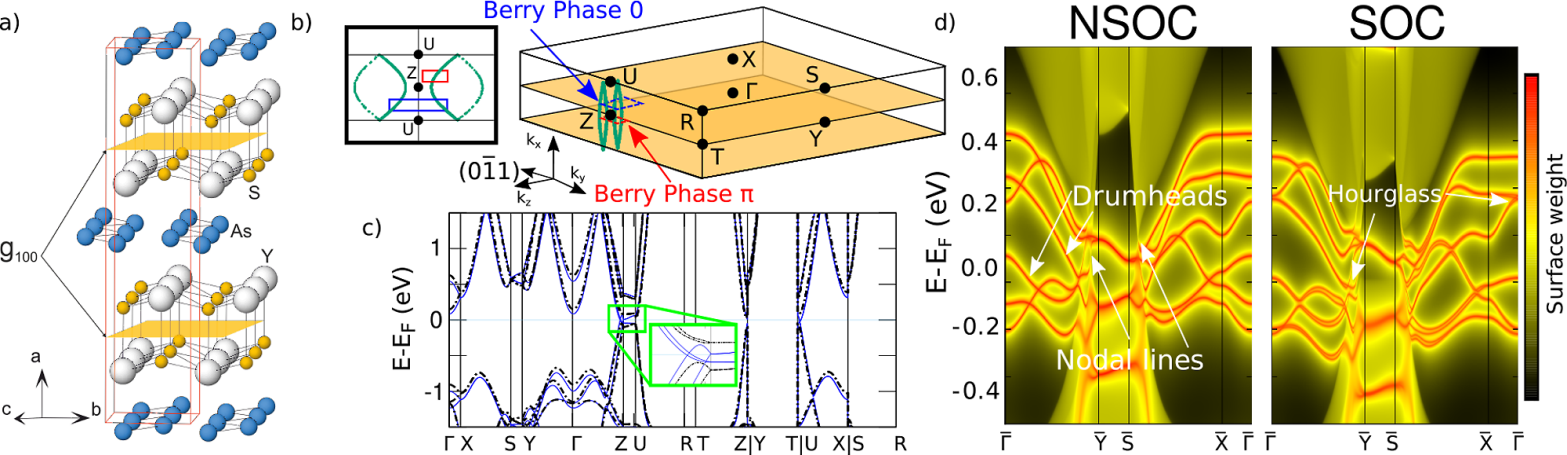
Bulk and surface properties of YAsS. (a) Crystal structure of YAsS in *Pnma* space group (#62), with the glide symmetry responsible for hourglass fermions displayed in orange. (b) BZ of orthorhombic *P* lattice. In orange, the plane in reciprocal space that is invariant under the action of the glide symmetry. In green, the nodal lines enforced by the *z*_2_^′^ = 1 topological invariant in the SOC-free phase. They are very close to each other, in neighboring BZs. We show the nodal lines in a projection parallel to *k*_*y*_ in the inset. In the Cartesian axis we show the reciprocal space direction in which hourglass states are predicted to exist in the surface. (c) Bulk band structure of YAsS without and with SOC. The inset shows the nodal line crossings in the absence of SOC. (d) Calculated (0–11) surface spectrum of YAsS. In the left, spinless surface spectrum. The white arrows point at the surface projection of bulk nodal lines, from which surface drumhead states emerge. In the right, spinful surface spectrum, showing the hourglass fermions.

**Table 1:** Irreducible Representations in the Absence and Presence of SOC at Γ and *U* K-points^a^

**K**	NSOC	SOC
Γ	4Γ_1_^+^⊕1Γ_1_^–^⊕2Γ_2_^+^⊕3Γ_2_^–^⊕ 2Γ_2_^+^⊕3Γ_2_^–^⊕4Γ_4_^+^⊕1Γ_4_^–^	12 ${\mathrm{\Gamma ̅}}^{+}⊕$ 8 ${\mathrm{\Gamma ̅}}^{-}$
*U*	4*U*_1_ *U*_4_^+^⊕2*U*_1_ *U*_4_^–^⊕1*U*_2_ *U*_3_^+^⊕3*U*_2_ *U*_3_^–^	5 ${\mathrm{U̅}}^{+}⊕$ 5 ${\mathrm{U̅}}^{-}$

a The band inversion happens at Γ, where there is an imbalance of even/odd eigenvalues in the irrep decomposition.

In the absence of SOC, it was shown that the SI $Z_{4}=2$ corresponds to topological invariant $z_{2}^{\prime }=\frac{Z_{4}}{2}=1$ ,^44^ which implies the presence of an odd number of topologically charged nodal lines in each half of the BZ (see Figure 2b). In our case, we find 1 nodal at each side of the *k*_*z*_ = π plane (see the *k*_*y*_ projection in Figure 2b).

The topological charge of these nodal lines is of $Z_{2}$ type, and it can be understood as the Berry phase along a loop that is ‘chained’ to the nodal line.^47^ In Figure 2b we show the path in momentum space that we used to determine the topological charge of one of the nodal lines (long dashed red line), with the resulting Berry phase of π. We similarly checked the other nodal line and we obtained the same result. To further confirm the $Z_{2}$ nature of the charge, we computed the Berry phase on a path that chains both nodal lines together (short dashed blue line) and obtained a Berry phase of 0, which is the result of the sum of the two *z*_2_^′^ = 1 topological invariants of both nodal lines. These nodal lines are expected to present surface states stemming out of their projection in the surface, similar to Fermi arcs stemming out of Weyl nodes, called drumhead states.^47–50^ We can see the drumhead states of YAsS in Figure 2d (left) in the (0–11) surface.

In the presence of SOC, the $Z_{4}=2$ SI implies the presence of glide and TRS protected surface states known as hourglass fermions,^51,52^ which are topological surface states whose characteristic “hourglass” shape is symmetry enforced. In Figure 2d we show the surface spectrum of YAsS, with the characteristic hourglass fermions. In Supporting Information we give further details on the origin of hourglass fermions and their symmetry protection.

#### Minimal Model

To clarify the emergence of the topological surface states and their robustness against electronic correlations, we construct a minimal model from the orbitals driving the band topology. Within this framework, the hourglass dispersion decomposes into an abstract layer construction consisting of two $Z_{2}=1$ two-dimensional topological insulators—one centered at the origin and the other at the unit-cell midpoint—coupled by a glide symmetry.^45^ In particular, one can observe in Figure 2a that As atoms are arranged in two layers—one at the origin and the other at the center of the unit cell—and in Figure 3a, the NSOC band structure of YAsS shows that the dominant weight near the Fermi level originates from the As p_*z*_ and p_*y*_ orbitals, as confirmed by the spatial distributions of the corresponding Wannier functions in Figure 3b. This implies that the minimal model can be constructed from As p_*z*_ and p_*y*_ orbitals located at those atomic positions.

**Figure 3:**
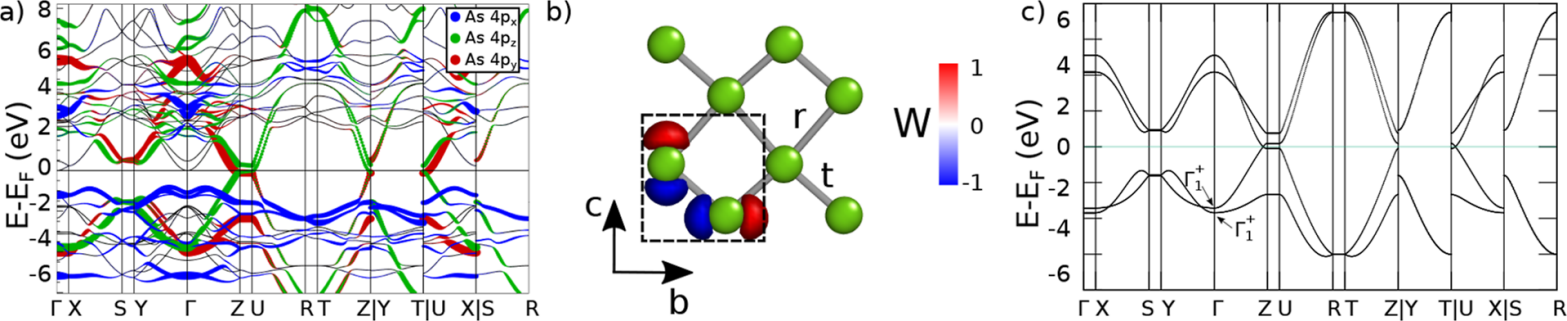
(a) Spinless bulk bands of YAsS, with orbital weights from p_*x*_, p_*y*_ and p_*z*_ orbitals of As. (b) Wannier functions from p_*z*_ and p_*y*_ orbitals at As sites in one layer. The color-scale shows the sign of the Wannier function. The hoppings t and r are the nearest neighbors hoppings of the minimal model. (c) Bulk bands of monolayer model. We can observe that there are two Γ_1_^+^ irreps in the valence bands, which produce the nontrivial $Z_{2}=1$ topological invariant.

This model will serve as a basis for explaining the basic topological properties of the compunds in the family that share the same orbital content close to the Fermi level. As we will see, this is the case for SmAsS, but not for LaAsS. For SmAsS, this construction can be extended to include the *f*-electron degrees of freedom stemming from Sm 4*f* orbitals. In what follows, we explicitly build this model first for the As monolayer and then for the As bilayer, which together form the full model.

#### As Monolayer $Z_{2}=1$

We construct maximally localized Wannier functions solely for As p_*z*_ and p_*y*_ orbitals from the NSOC DFT calculation, and we keep only first nearest neighbors of the interpolated Hamiltonian. This model is depicted in Figure 3b, the bulk band structure is shown in Figure 3c and full details on Wyckoff position and numerical values of the hoppings can be found in Supporting Information. Using the Mathematica package MagneticTB,^53,54^ we compute the inversion eigenvalues of the monolayer model at half filling, confirming a $Z_{2}=1$ topological invariant. Figure 3c presents the irreducible representations that drive the band inversion, reproducing the minimal inversion pattern of the full system. By combining this invariant with the absence of SOC, the system is forced into a semimetallic phase, exhibiting two Dirac crossings at (*k*_*y*_, *k*_*z*_) = ± (0, 0.753)­Å (see Figure 4a). Since the 2D Dirac points can be viewed as flat nodal lines along *k*_*x*_, their surface states are analogous to the drumhead states that emerge from projecting 3D nodal lines.^47^

**Figure 4:**
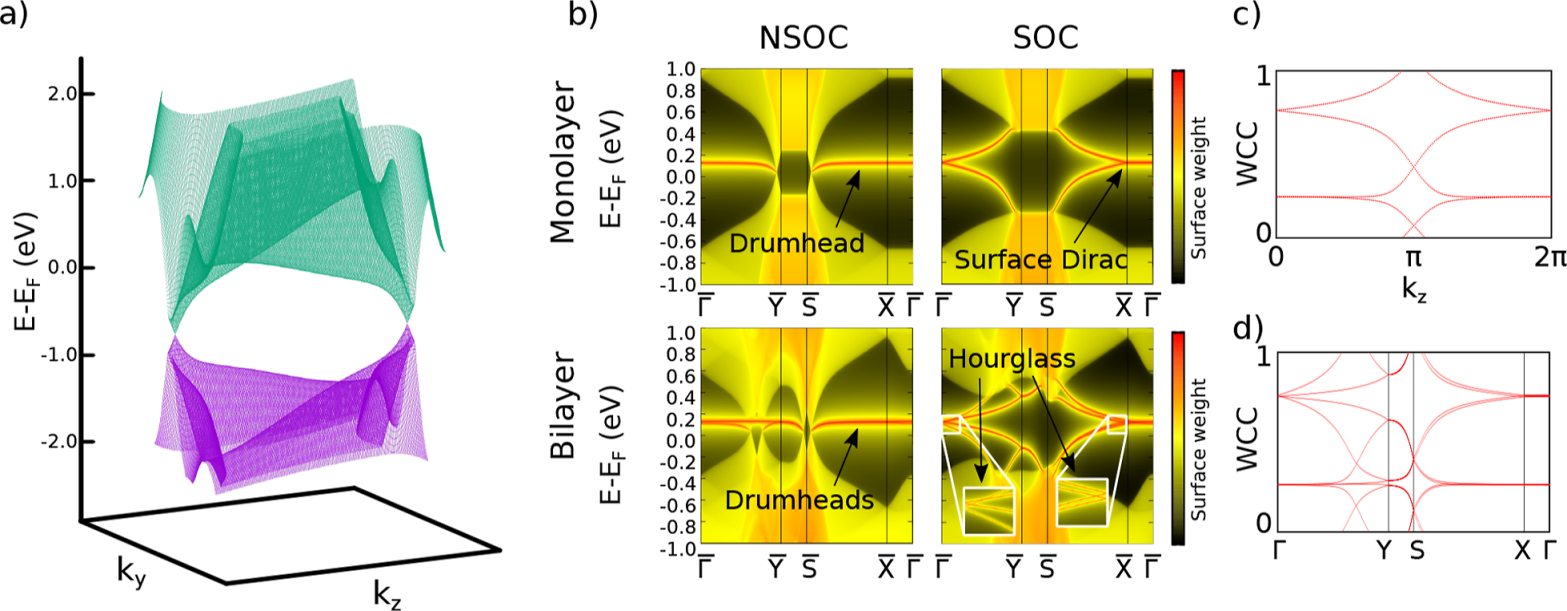
Bulk and surface properties of the monolayer and bilayer models. (a) Bulk band dispersion of last valence and first conduction bands of the monolayer model, showing the two Dirac crossings. (b) Surface spectrum of the models. In the top row, we show the monolayer surface spectrum without and with SOC. In the bottom row, we show the bilayer surface spectrum, without and with SOC. For illustrative purposes, we increased the hoppings by *a* factor of 100 in the plots. Wilson loops of the monolayer (c) and bilayer (d) models.

We incorporate spin–orbit coupling via the dominant Wannier term, *H*_SOC_ = λ*i*σ_*y*_, acting on-site between the p_*y*_ and p_*z*_ orbitals. To open a substantial gap and clearly resolve the surface states, we set λ = 0.5 eV. This term gaps the Dirac nodes while leaving the occupied irreducible representations unchanged, so the $Z_{2}$ invariant is preserved.

To further validate this result, we compute the Wilson loops by integrating along *k*_*y*_ and plotting versus *k*_*z*_ (see Figure 4c), which exhibit the characteristic winding of a $Z_{2}=1$ 2D TI. We also calculate the surface spectrum, revealing the expected surface Dirac nodes (see Figure 4b, top-right panel).

#### As Bilayer: $Z_{4}=2$

Starting from the monolayer tight-binding Hamiltonian, we build the full (bilayer) model by appending a second monolayer related via the glide symmetry *g*_100_. All intralayer hopping amplitudes are left unchanged, while the interlayer couplings are then obtained by extracting nearest-neighbor hopping parameters from the Wannierization. In the absence of SOC, surface states originating from both nodal lines emerge (Figure 4b, bottom left). Upon introducing SOC, a gap opens and these surface states split, yielding the characteristic hourglass-Fermion dispersion (Figure 4b, bottom right). To verify this, we compute the Wilson loop spectrum integrated along *k*_*x*_ following the same surface momentum path as in Figure 4b; the resulting bands (Figure 4d) exhibit the expected winding for a $Z_{4}=2$ topological invariant.^51^

In summary, this minimal model faithfully reproduces all of YAsS’s topological features at the Fermi level. Furthermore, because SmAsS exhibits a comparable orbital character at Fermi level, the same model can serve as an effective proxy for other members of the family with the same orbital content.

### Electronic Structure of SmAsS

To explore the interplay between topology and *f*-electron–driven heavy-fermion physics, we extend the minimal model developed in previous section to SmAsS. We carried out density functional theory (DFT) calculations using the meta–Becke–Johnson potential, which has been demonstrated to reliably reproduce electronic band gaps.^55^ The resulting bulk band structure is shown in Figure 5a, with the Sm 4*f* orbital character highlighted in purple at the Fermi level. Experimentally, we estimated the value of the Sommerfeld coefficient, which reflects the effective electron mass (see Figure 1e), in agreement with the flat-band dispersion predicted by our DFT results. The As 4*p* orbital weights remain essentially unchanged from those in YAsS (see Figures 3a and 5a). Moreover, we perform DFT calculations of SmAsS setting the Sm 4*f*-electrons in the core (see Figure 5b) and we find the orbital weight of As 4*p* orbitals remains unchanged too. Consequently, introducing *f*-electrons does not affect the band inversion at the Γ point that gives rise to the nontrivial topology. Instead, *f*-electron occupancy shifts the topological gap downward by roughly 0.4 eV—an effect we verify through calculated SIs. A comparable topological phase also appears about 1 eV above the Fermi level.^22,23^ While the 4*f* orbitals do not invert with As *p*-orbitals, their renormalization of the energy scale enhances the topological gap’s spectroscopic visibility for experimental techniques such as angle-resolved photoemission spectroscopy (ARPES).

**Figure 5:**
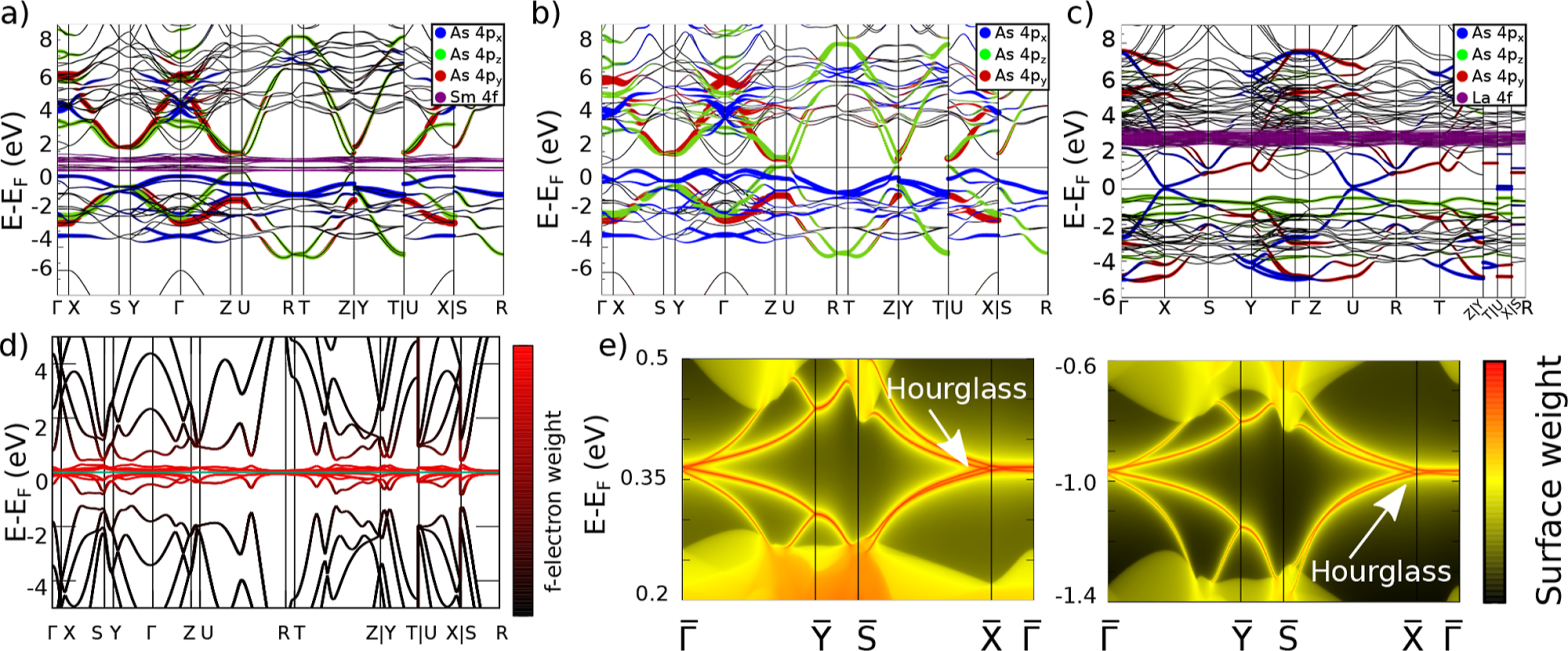
Bulk and surface spectrum including the *f*-electrons. (a) Bulk bands of SmAsS. In color, the orbital weight of As 4*p*_*i*_ orbitals (*i* = *x*, *y*, *z*) and Sm 4*f* orbitals. (b) Bulk bands of SmAsS with the Sm 4*f*-electrons in the core. We can see how the orbital weight of As 4*p* orbitals is identical. (c) Bulk band structure of LaAsS. Notice the difference in the orbital weight close to the Fermi level compared to Y/SmAsS. (d) Bulk band structure of the heavy-fermion model. The colorbar represents the weight of the *f*-orbitals. (e) Surface spectrum of the heavy-fermion model in both topological gaps.

Our DFT calculation yields an average occupation of 5.33 electrons in the 4*f*-shell of Sm. This number lies between the fillings expected for the Sm^+3^ (5 electrons) and Sm^+2^ (6 electrons) oxidation states, which indicates that SmAsS is a mixed-valence insulator as for our DFT analysis. This result is consistent with previous theoretical and experimental reports of the valence of Sm in SmB_6_.^17,56–61^ Furthermore, the small hybridization between the Sm 4*f* states and the rest of bands observed in Figure 5a might favor the stabilization of the intermediate occupancy against electronic correlations. Of course, a more detailed analysis regarding the valence of Sm in SmAsS will be possible via spectroscopic investigations,^62,63^ which are currently underway. Moreover, the Kondo insulator picture also sets the stage for SmAsS to be studied for other associated correlation features, such as midgap states, especially in the inevitable presence of defects in the system, both theoretically and experimentally.

#### Anderson Lattice Model

Since the orbital weight of As 4*p* orbitals in SmAsS, both taking into account explicitly the *f*-electrons and placing them on the core, is very similar to that of YAsS, we can extend the model derived previously to account for the *f*-electrons. This will allow us to probe the robustness of the topology and validate our DFT results. Specifically, we employ an Anderson lattice Hamiltonian with itinerant As-derived electrons and strongly correlated Sm *f*-electrons respecting the symmetries of space group *Pnma* (No. 62). We stress that space group symmetry constraints play a central role in our treatment of the strong correlation effects.

The Hamiltonian of the itinerant electrons is $H_{c}=\sum_{k}\Psi_{k}^{†}\left(h\left(k\right)-\mu \right)\Psi_{k}$ where μ is chemical potential, *h*(**k**) is the single-particle minimal model described above, and Ψ=(*c*_*q*_1_↑_,*c*_*q*_1_↓_,...,*c*_*q*_8_↑_,*c*_*q*_8_↓_) is the annihilation operator of itinerant electrons at As sublattice sites with spin degrees of freedom.

We assume the *f*-electrons in the Sm atoms are at the energy level ϵ_*f*_ and consider a Hubbard interaction in the infinite *U* limit.^64^ Here the system is effectively described by an auxiliary-boson representation. This model is solved at the saddle-point level, which formally develops in a large*N* limit.^22,64,65^ The effective Hamiltonian is given as follows

2 $$\begin{array}{l} H=H_{c}+\sum_{j,\alpha ,\sigma }\epsilon_{f}f_{j\alpha \sigma }^{†}f_{j\alpha \sigma }+rV\left[c_{j\alpha \sigma }^{†}f_{j\alpha \sigma }+h.c.\right]\\ +\lambda \sum_{j}\left(n_{f,j}+8r^{2}-Q\right) \end{array}$$

where *j*, α and σ stand for unit cell position, sublatttice and the spin degrees of freedom, respective. The factor 8 before *r* ^2^ refers to the number of sublattices in each unit cell. The *f*-electron particle number $n_{f,j}=\sum_{\alpha \sigma }f_{j\alpha \sigma }^{†}f_{j\alpha \sigma }$ is constrained by the Lagrange multiplier λ term and $r=\sqrt{Z}$ is the square-root of the quasi-particle weight. A nonzero value of quasiparticle weight indicates the emergence of the Kondo resonance, which appears in the vicinity of the Fermi energy. We choose ϵ_*f*_ = −1, *V* = 1, μ = 0, and *Q* = 8 to maintain the half filling constraint on each sites. The orbital-resolved bulk band structures and the corresponding surface spectrum are shown in Figure 5d,e, respectively.

The *f*-electron level is pinned to the Fermi level due to the Kondo effects, which place the *f*-electron excitations to be tied to the Fermi level within the Kondo energy scale, along with the half filling constraint. From the hybridization with the *p*-orbital bands, the *f* bands split and move the topological gap; one above and one below the Fermi level, maintaining the $Z_{4}=2$ topological invariant. The model confirms the DFT prediction that the localized *f*-electrons do not destroy the topological gaps, but move them in energy. The results at the model calculation level are robust even when further fluctuation effects are incorporated, so long as the quasiparticle picture applies as expected for such lattice systems without a deliberate tuning to the quantum critical regime. In addition, while the model can be generalized to include even more realistic features and additional interaction terms, the essential physics should already be captured by our model calculation given that we have fully captured the crystalline symmetry constraints.

### Electronic Structure of LaAsS

Our experimental results reveal a new crystal structure for LaAsS, distinct from that previously reported.^30^ While it crystallizes in space group *Pnma*, like Y/SmAsS, we observe a $\sqrt{2}\times \sqrt{2}$ supercell perpendicular to the long axis. Figure 1c compares the structures of LaAsS and SmAsS, highlighting the distortion and rotation of the As lattice in LaAsS relative to SmAsS.

Figure 5c presents the bulk band structure of LaAsS, calculated using the meta–Becke–Johnson potential. Due to the structural distortion, maintaining the conventional *Pnma* notation requires assigning the long-axis direction (out-of-plane) along *x* for Y/SmAsS and along *z* for LaAsS. In both compounds, the out-of-plane orbitals (*p*_*y*_ for Y/SmAsS, *p*_*z*_ for LaAsS) lie away from the Fermi level. However, the in-plane orbital character differs significantly: while both *p*_*x*_ and *p*_*z*_ contribute in Y/SmAsS, only *p*_*x*_ contributes in LaAsS.

Following the analysis for Y/SmAsS, we computed the irreducible representations at Γ and *U* in order to extract the $Z_{4}$ SI. We show the irreps in Table 2. Compared to Y/SmAsS, it presents the same band inversion at the Γ point. However, there is an extra band inversion at the *U* point, which results in a $Z_{4}=0$ SI, thus rendering the system trivial. We repeated the calculation on the unoptimized structure, that is, the experimentally determined structure, which results in a reordering of the irreps at *U* and a $Z_{4}=2$ SI, as Y/SmAsS. Since the gap of LaAsS is very small, we expect it to be very sensitive both to modeling (DFT calculations with different exchange–correlation approximations) and experimental conditions (external pressure, strain or growing conditions). In order to test this, we run calculations for the strained structure. Taking as a reference the unstrained relaxed structure, we introduced uniform compressive and expansive strain of 2, 4 and 6%. Fixing the cell, we let the atomic positions relax. We found that for the expansive strains the system remains topologically trivial, while all compressive strains (2, 4 and 6%) result in a band inversion at the *U* point and symmetry indicator $Z_{4}=2$ (see Figure 6). There is a band inversion at the *X* point as well. However, the little cogroup at the *X* point contains only one irrep, so the topology remains unchanged by any band inversion at the *X* point.

**Table 2:** Irreducible Representations of LaAsS at Γ and *U* K-points^a^

***K***	Irreps
Γ	12 ${\mathrm{\Gamma ̅}}^{+}⊕$ 8 ${\mathrm{\Gamma ̅}}^{-}$
*U* (optimized)	4 ${\mathrm{U̅}}^{+}⊕$ 6 ${\mathrm{U̅}}^{-}$
*U* (experimental)	5 ${\mathrm{U̅}}^{+}⊕$ 5 ${\mathrm{U̅}}^{-}$

a The band inversion at γ remains, but there is an extra band inversion at *U*, which renders the system trivial.

**Figure 6:**
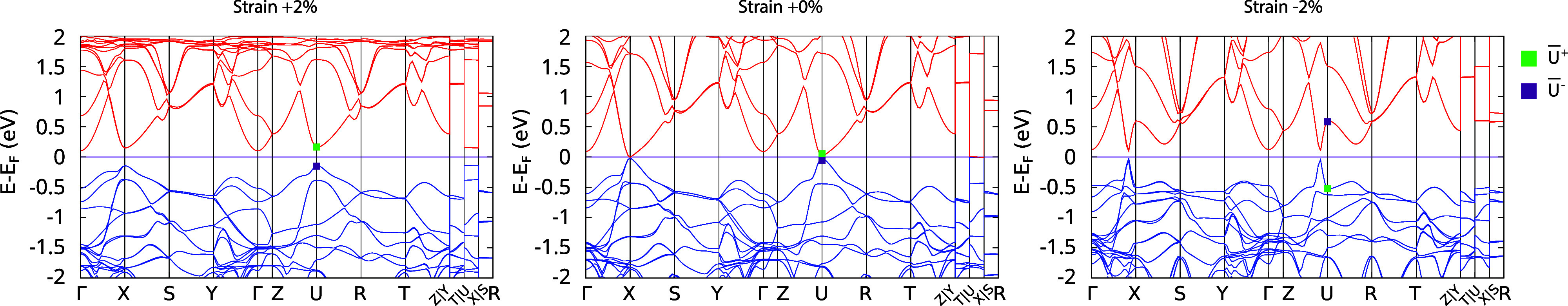
Bulk bands of homogeneously strained LaAsS. Notice the band inversion at the *U* point as a function of compressive strain.

## Conclusions

We have identified *R*AsS (*R* = Y, La, Sm) as a new family of topological crystalline insulators featuring glide-symmetry-protected hourglass fermions. Our structural analysis clarified their accurate crystal structure and our analysis based on DFT and TQC revealed nontrivial topological invariants in YAsS and SmAsS, with LaAsS near a topological transition. We show that both YAsS and SmAsS present topologically protected surface states in the surface preserving the glide symmetry. SmAsS stands out as an example of *f*-electron and topology interplay. We demonstrate that moderate *f*-electron correlations can create correlated electron excitations near the Fermi level and, thus, shift and renormalize topological features without destroying symmetry protection. Density functional theory and tight-binding calculations show that their topological properties can be captured with a minimal model. Crucially, in SmAsS, the surface states persist despite *f*-electron interactions and shift downward in energy, enhancing their accessibility for spectroscopic studies, such as ARPES. While correlation is expected to cause spectral broadening, the fact that the surface states are not accompanied by lower-energy electronic bulk or surface states—due to the insulating nature of the system—leads to the expectation that the spectral broadening will be relatively small. This, along with the topological protection, suggest both the robustness and experimental accessibility of the surface states. These results expand the landscape of strongly correlated topological insulating materials beyond the two reported ones (SmB_6_ and YB_12_), while unveiling a new mechanism underlying such materials in which symmetry constraints work in tandem with strong correlations, and offer a platform for studying interaction-driven phenomena.

## Methods

### Synthesis and Characterization

Preparation and synthesis took place in a glovebox (O_2_, H_2_O ≤ 0.1 ppm). Glassy carbon crucibles were heat-treated under a dynamic vacuum before use. The starting materials were: yttrium pieces (ChemPur, 99.9%), lantanum chunks (Alfa Aesar, 99.9%), samarium powder (ChemPur, 99.9%), arsenic lump (Alfa Aesar, Puratronic, 99.9999%), sulfur pieces (Alfa Aesar, 99.999%). Powdered LiCl (Thermo Scientific, Ultra Dry, 99.9%) and RbCl (Thermo Scientific, 99.8%) with a ratio of 1:1 were used as a flux. Prior to synthesis, the salts were preheated inside the glovebox to remove moisture. All starting materials with the total mass of 1 g for *R*, As, and S (ratio 1:1:1), and 2.5 g of salt mixture (ratio 1:1) were loaded in a glassy carbon crucible, covered by flux material, and enclosed in a Ta ampule using the arc-welding machine. Ta ampule with glassy carbon crucible was placed in a vertical furnace and treated with the following temperature profile—heated over 12 h to 900 °C, kept for 168 h at this temperature, and cooled down to room temperature over 240 h. The cooling rate is ∼4° per hour. Residual salt was washed away with deionized water. Typical crystals have dimensions on the order of 20 × 20 × 2 μm^3^. The crystals appear to be stable in air.

The lattice parameters were established by using high resolution powder diffraction data (λ = 0.35466 Å) recorded at ID22 beamline at European Synchrotron Radiation Facility (ESRF). For single crystal experiments, small pieces were used (∼20 μm). The single crystal diffraction data were collected using a Rigaku AFC7 diffractometer (equipped with a Saturn 724+ CCD detector) and a Bruker Apex II diffractometer, both with Mo Kα radiation (λ = 0.71073 Å). The complete crystallographic information is given in Tables S1–S3.

The single crystals of YAsS, LaAsS and SmAsS were additionally analyzed by energy-dispersive X-ray spectroscopy with a Jeol JSM 6610 scanning electron microscope equipped with an UltraDry EDS detector (ThermoFisher NSS7). The semiquantitative analysis was performed with 30 keV acceleration voltage. No impurity phases were observed, confirming that no reaction with the crucible took place during synthesis. The experimentally determined element ratios were in a good agreement with the 1:1:1 stoichiometry.

Temperature- and field-dependent magnetic measurements were conducted in a Quantum Design (QD) Magnetic Properties Measurement System (see Figure 1). Several single crystals of YAsS and SmAsS were mounted on a quartz capillary. Magnetic properties were measured at temperatures ranging from 2 to 300 K and in magnetic fields up to *H* = 7 T. The specific heat data were collected on a QD Physical Property Measurement System (PPMS) from *T* = 0.4 K to *T* = 100 K in *H* = 0 and *H* = 9 T magnetic fields. For measurements of electrical resistivity, microscale devices were fabricated out of a YAsS, SmAsS or LaAsS single crystal by using a plasma focused-ion-beam (FIB). AC electrical resistivity measurements were performed by a QD PPMS, using a standard four-probe technique at temperatures between *T* = 2 and 300 K in *H* = 0 and *H* = 9 T applied magnetic field. A current pulse of 0.01 mA with frequency 93 Hz for 1 s was applied.

### Calculations

We performed DFT calculations as implemented in the Vienna ab initio simulation package (VASP)^66–69^ for structural optimization and determination of symmetry indicators. The interaction between the ion cores and valence electrons was treated by the projector augmented-wave method,^70^ the generalized gradient approximation (GGA) was employed for the exchange–correlation potential with the Perdew–Burke–Ernzerhof for solid parametrization,^71^ and the spin–orbit coupling (SOC) was considered based on the second variation method.^72^ A Γ-centered Monkhorst–Pack *k*-point grid of (5 × 9 × 9) was used for reciprocal space integration for Y/SmAsS and (9 × 9 × 5) for LaAsS, and 500 eV energy cutoff of the plane-wave expansion. We ensured convergence up to 10^–5^ eV per unit cell. We performed geometric optimization of the structure until the forces in the atoms were smaller than 10^–2^ eV/Å. We computed the irreducible representations of the occupied set of bands in all systems using Vasptotrace software and computed the SIs as implemented in the Bilbao Crystallographic Server.^73–75^ To enforce symmetry constraints, we constructed maximally localized Wannier functions from a DFT calculation as implemented in FPLO,^76^ and we run surface state calculations following the iterative Green’s function method as implemented in WannierTools.^77^

Equation 2 is solved at the saddle point level, where the self-consistent equations are derived by taking the partial derivatives of the effective Hamiltonian with respect to λ and *r*, respectively. It leads to

3 $$\left\langle n_{f,j}\right\rangle +8r^{2}=Q$$

4 $$V\sum_{\alpha ,\sigma }\left\langle c_{j\alpha \sigma }^{†}f_{j\alpha \sigma }\right\rangle =-16\lambda r$$

where angle brackets ⟨·⟩ means averaging over all sites in the unit cell. These equations are solved on a 40 × 40 × 10 grid. We find *r* = 0.805, λ = 0.503 for the parameters we adopted.

## Supplementary Material



## Notes

Supporting Information contains the tables with detailed crystallographic information on the three compounds we report. It also contains the analysis of specific heat. There is also an expansion of the surface spectrum implications of the $Z_{4}=2$ symmetry indicator as well as the necessary details to construct of the minimal tight binding model (PDF)
