# Supplementary material for: Emergent Heavy-Fermion Physics in a Family of Topological Insulators RAsS (R = Y, La, and Sm)
Source: J Am Chem Soc. 2026 Mar 10;148(12):12979–89. doi: 10.1021/jacs.5c21743 (PMC13047537; doi:10.1021/jacs.5c21743)
Supplement: Supplementary file 1 [file ja5c21743_si_001.pdf]

Emergent heavy-fermion physics in a family of topological insulators RAsS (R = Y, La, and Sm) Supporting Information

Iñigo Robredo*,*,† Yuan Fang,‡ Lei Chen,‡ Nazar Zaremba,§ Yurii Prots,§ Mitja Krnel,§ Markus König,§ Mikel I. Iraola,‖ Thomas Doert,⊥ Jeroen van den Brink,‖ Claudia Felser,§ Qimiao Si*,*,‡ Eteri Svanidze*,*,§ and Maia G. Vergniory**,#

†*Luxembourg Institute of Science and Technology (LIST), Avenue des Hauts-Fourneaux 5, L-4362 Esch/Alzette, Luxembourg*

‡*Department of Physics & Astronomy, Extreme Quantum Materials Alliance, Smalley-Curl Institute, Rice University, Houston, Texas 77005, USA*

¶*Department of Physics and Astronomy, Stony Brook University, Stony Brook, NY 11794, USA*

§*Max Planck Institute for Chemical Physics of Solids, Dresden 01187, Germany*

‖*Institute for Theoretical Solid State Physics, IFW Dresden, 01069 Dresden, Germany*

⊥*Technical University of Dresden, Dresden 01062, Germany*

#*Donostia International Physics Center, 20018 Donostia-San Sebastián, Spain*

@*Département de Physique et Institut Quantique, Université de Sherbrooke, Sherbrooke, J1K 2R1, Québec, Canada.*

△*Regroupement Québécois sur les Matériaux de Pointe (RQMP), Quebec H3T 3J7, Canada*

E-mail: inigo.robredo@list.lu; qmsi@rice.edu; svanidze@cpfs.mpg.de; maia.vergniory@usherbrooke.ca

Crystallographic information

TABLE S 1: Crystallographic data of YAsS, SmAsS, and LaAsS (standard setting).

Composition	YAsS	SmAsS	LaAsS
Structure type	SmAsS	SmAsS	GdPS
Space group		*Pnma*	
Pearson symbol	*oP*12	*oP*12	*oP*24
Formula units per unit cell, Z	4	4	8
Unit cell parameters[1]			
a, Å	16.88916(6)	17.1455(2)	5.7078(6)
b, Å	3.80572(1)	3.86008(3)	5.6924(5)
c, Å	3.84082(1)	3.91298(3)	17.553(2)
Unit cell volume, $Å^3$	246.870(1)	258.973(4)	570.32(8)
Calculated density, g/cm^{-3}	5.29	6.60	5.73
Diffraction system	Bruker D8	Bruker D8	RIGAKU AFC7
Detector	Apex II CCD	Apex II CCD	Saturn 724+ CCD
Radiation λ Å		MoKα, 0.71073	
Scan; step / degree; N(images)	0.3, 4934	0.3, 5992	ϕ, 0.5, 720
Maximal 2θ / degree	80.35	80.30	66.23
Measured range in *hkl*	$-30 \leq h \leq 30$	$-31 \leq h \leq 31$	$-3 \leq h \leq 8$
	$-6 \leq k \leq 6$	$-7 \leq k \leq 6$	$-8 \leq k \leq 8$
	$-6 \leq l \leq 6$	$-7 \leq l \leq 7$	$-26 \leq l \leq 24$
Absorption correction		multi-scan	
T(max)/T(min)	2.13	2.20	3.95
Absorption coefficient, mm^{-1}	37.4	35.8	26.9
N(*hkl*) measured	15720	14949	4727
N(*hkl*) unique	921	952	1224
R(int)	0.0566	0.0438	0.0162
N(*hkl*) observed	885	935	1143
Observation criteria		F(*hkl*) $\leq 4\sigma$ [F(*hkl*)]	
Twinning law	-100	-100	010
	0 0 1	0 0 1	1 0 0
	0 1 0	0 1 0	0 0 -1
Twin components ratio	0.518(2):0.482	0.688(3):0.312	0.929(4):0.071
Number of refined parameters	22	22	35
R1	0.0240	0.0282	0.0339
wR2	0.0652	0.0722	0.0969
Residual peaks (e^- $Å^{-3}$)	2.04/-2.47	2.53/-4.72	2.53/-1.98

TABLE S 2: Atomic coordinates and equivalent displacement parameters (in Å^2) in the YAsS, SmAsS and LaAsS (standard setting).

Atom	Wyckoff site	x/a	y/b	z/c	Ueq.
		YAsS ($Pnma$, SmAsS structure type)			
Y	4c	0.14574(2)	¼	0.23305(9)	0.00391(10)
As	4c	0.00146(3)	¼	0.71659(11)	0.00427(10)
S	4c	0.31334(5)	¼	0.2357(2)	0.00395(14)
		SmAsS ($Pnma$, SmAsS structure type)			
Sm	4c	0.14572(2)	¼	0.23234(8)	0.00481(9)
As	4c	0.00172(5)	¼	0.71092(19)	0.00576(12)
S	4c	0.31399(8)	¼	0.2344(4)	0.0054(2)
		LaAsS ($Pnma$, GdPS structure type)			
La1	4c	0.01102(7)	¼	0.35162(2)	0.00856(14)
La2	4c	0.48941(7)	¼	0.64314(2)	0.00897(14)
As	8d	0.28045(11)	0.02824(10)	0.00131(2)	0.01224(15)
S1	4c	0.0089(3)	¼	0.18347(11)	0.0089(3)
S2	4c	0.4908(3)	¼	0.81282(11)	0.0092(3)

TABLE S 3: Anisotropic displacement parameters (in Å^2) for YAsS, SmAsS and LaAsS (standard setting).

Atom	U_{11}	U_{22}	U_{33}	U_{23}	U_{13}	U_{12}
		YAsS ($Pnma$, SmAsS structure type)				
Y	0.00198(12)	0.0083(3)	0.00148(18)	0	0.00008(8)	0
As	0.00155(14)	0.0090(2)	0.00231(16)	0	-0.00025(10)	0
S	0.0015(3)	0.0085(6)	0.0018(5)	0	-0.00018(19)	0
		SmAsS ($Pnma$, SmAsS structure type)				
Sm	0.00292(12)	0.00524(19)	0.00628(18)	0	0.00014(7)	0
As	0.0029(2)	0.0063(3)	0.0081(3)	0	0.0001(2)	0
S	0.0033(4)	0.0058(7)	0.0070(7)	0	0.0001(3)	0
		LaAsS ($Pnma$, GdPS structure type)				
La1	0.0086(2)	0.0077(2)	0.0094(2)	0	0.00022(10)	0
La2	0.0088(2)	0.0076(2)	0.0105(2)	0	-0.00004(10)	0
As	0.0136(3)	0.0127(3)	0.0105(3)	0.00106(19)	0.00104(14)	-0.00021(17)
S1	0.0091(7)	0.0080(7)	0.0098(7)	0	-0.0005(5)	0
S2	0.0097(7)	0.0080(7)	0.0100(7)	0	0.0004(5)	0

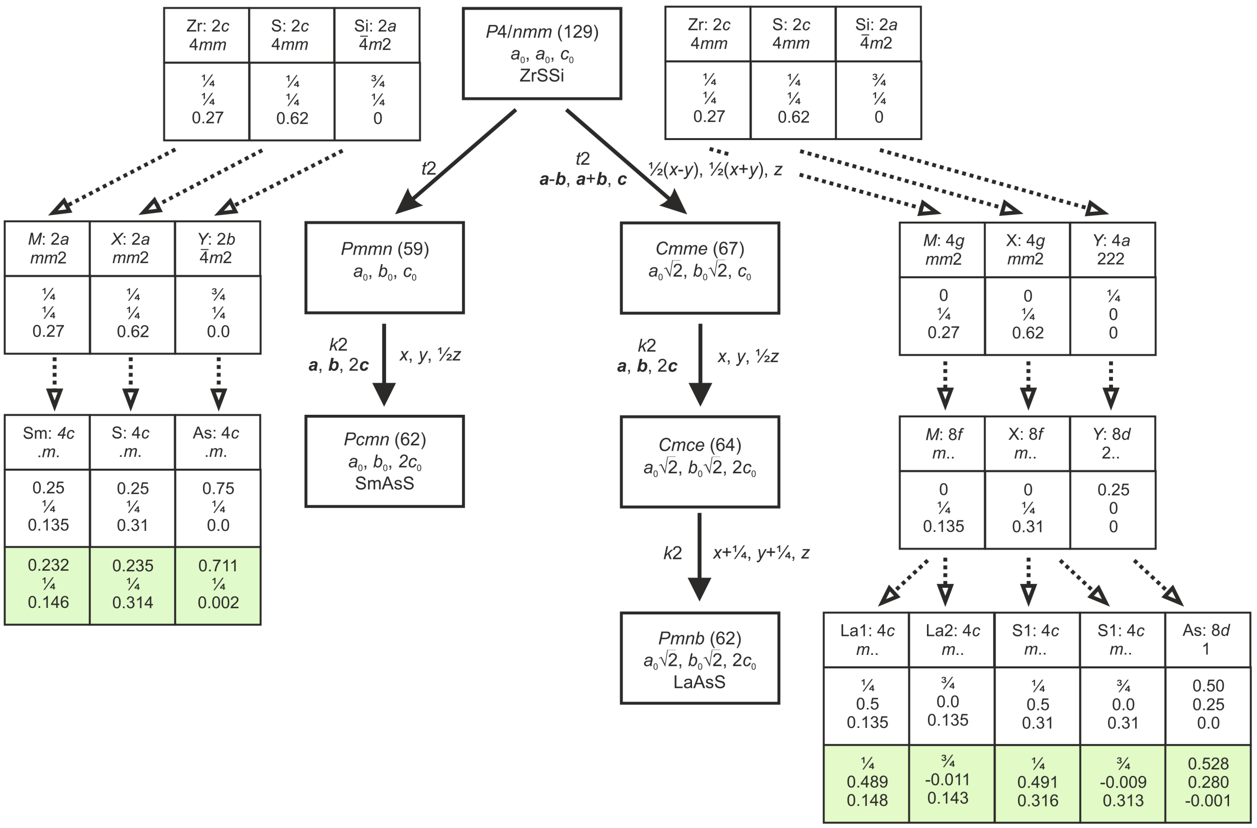

Figure 1: The Bärnighausen trees for LaAsS and SmAsS, showing their relation to the ZrSiS structure type; the atomic coodinates of LaAsS and SmAsS (white boxes: predicted, green boxes: experimental) can directly be derived by the symmetry reduction. Note that since it is not possible to have the tree in all-standard setting – the viewing directions in the tetragonal and orthorhombic crystal system are different. The calculations haven been done using the standard *Pnma* setting of the space group types.

Specific heat analysis

The analysis of specific heat is frequently used to investigate heaviness of a given system.[14–16] At low temperatures, the specific heat can be described by the Debye law: $C_p = \gamma T + \beta T^3$ (alternatively $\frac{C_p}{T} = \gamma + \beta T^2$), where the coefficients γ and β – also known as Sommerfeld coefficient and lattice specific heat – can be extracted from a linear fit of C_p/T vs. T^2 (see main text Fig. 1d). For systems not showing any transitions, the fit should be done at the lowest possible temperature, but in the case of a superconducting or magnetic transition the fit above transition temperature can be used to estimate the value of the Sommerfeld coefficient, which reflects the effective electron mass (since below the transition, magnetic or superconducting contribution(s) to the specific heat prevent an accurate assessment of the electronic contribution.[15,16]) This type of analysis is considered valid for temperatures below $\Theta_D/50$ or even $\Theta_D/10$.[16] In our case, the fitting at very low temperatures is not possible due to the magnetic transition at 7.5 K. We therefore fit in the range of $T = 10 - 20$ K. The fit is linear with $R^2 = 0.99824$, and we extract $\gamma = 160$ mJ $\text{mol}_{\text{Sm}}^{-1}$ K^{-2}, $\beta = 73.9$ μJ/mol K^4 for SmAsS. From these values, we can estimate the Debye temperature Θ_D for SmAsS (as extracted from the following equation:[40]

$$\theta_{\text{D}} = \sqrt[3]{\frac{14\pi^4 N_{\text{A}} r k_{\text{B}}}{5\beta}}, \tag{1}$$

where $r = 3$ is the number of atoms per formula unit in SmAsS and β is extracted from our fit. We estimate the Debye temperature $\Theta_D = 420$ K. This validates our choice of temperature range for fitting. It is important to note that this value of γ is consistent with an enhanced electron mass of SmAsS; for normal metals, γ is typically on the order of a few mJ $\text{mol}_{\text{Sm}}^{-1}$ K^{-2} – see, for example $SmMg_2Bi_3$[41] with $\gamma = 2$ mJ $\text{mol}_{\text{Sm}}^{-1}$ K^{-2} and $SmCu_2$[42] with $\gamma = 6$ mJ $\text{mol}_{\text{Sm}}^{-1}$ K^{-2} extracted using the same method.

Implications of $\mathbb{Z}_4 = 2$ symmetry indicator and surface spectrum

In this appendix, we illustrate the symmetry protection of hourglass fermions in space group $Pnma$. The glide symmetry g_{100} leaves the planes $k_x^p = 0, \pi$ invariant. We can get the eigenvalues of this symmetry at a general k-point (k_x^p, k_y, k_z) by noticing that:

$$\left(\{m_x|\frac{1}{2}\frac{1}{2}\frac{1}{2}\}\right)^2 = \{E|011\} = R_y + R_z, \tag{2}$$

where $R_y + R_z$ is an integer lattice translation in the yz direction. Restricting to the $k_z = 0$ plane, the eigenvalues of the glide are $\pm\sqrt{e^{ik_y}} = \pm e^{ik_y/2}$. This is also true for the states in the $(01\bar{1})$ surface Brillouin zone in the $\bar{\Gamma} - \bar{Y} = (0, \bar{k}_y)$ and $\bar{X} - \bar{S} = (\pi, \bar{k}_y)$ paths. Focusing on the first path, we compute the eigenvalues of surface bands at both extremes. At $\bar{\Gamma} = (0,0)$, the eigenvalues are $\pm e^{i0/2} = \pm 1$. Due to time reversal symmetry-enforced Kramers degeneracy, the bands at $\bar{\Gamma}$ are doubly degenerate, both of which have to share either $+1$ or -1 glide eigenvalue. At the other extreme, at $\bar{Y} = (0, \pi)$, the eigenvalues of the glide symmetry are $\pm e^{i\pi/2} = \pm i$. In this case, TRS maps the state with eigenvalue $+i$ to the state with eigenvalue $-i$, thus, Kramers pairs need to have opposite glide eigenvalues. Since the glide is a symmetry of the whole line, we can follow the bands from $\bar{\Gamma}$ to $\bar{Y}$ by labeling the eigenvalues. Once away from the $\bar{\Gamma}$ point, TRS is not a symmetry of the little group and thus Kramers pairs will split. In particular, the states that emerge from eigenvalue $+1$ (-1) will evolve to eigenvalue $+e^{ik_y/2}$ $(-e^{ik_y/2})$. At the end of the path, at $\bar{Y}$, the states with eigenvalue $+e^{ik_y/2}$ $(-e^{ik_y/2})$ will have eigenvalue $+i$ $(-i)$. Since TRS forces Kramers pairs to have opposite eigenvalues, the Kramers partners must change along the path, i.e., the bands *cross* along the path. Notice that bands that must cross carry different eigenvalues in the path, so their crossing is glide-symmetry protected. This procedure holds for the $\bar{X} - \bar{S}$ path as well. For the other two high-symmetry paths, i.e. $\bar{\Gamma} - \bar{X}$ and $\bar{Y} - \bar{S}$, the little group of the line contains the combination of the glide and TRS, so that the bands are Kramers-degenerate along the whole line. A sketch of the discussion is shown in Fig. 2a and

Fig. 2b. Notice that this result only holds if both TRS and the glide symmetry are present in the surface termination. Even if it seems unrealistic to obtain such a symmetric surface, the symmetry needs to be respected only approximately, as is shown in recent experimental works.[52]

TABLE S 4: Numerical values of the minimal monolayer TB model. All terms are written in units of eV.

	p_z-p_z (eV)	p_z-p_y (eV)	p_y-p_y (eV)
on-site	0.0	-	0.264
t	0.851	1.949	1.325
r	0.981	1.368	0.671

TABLE S 5: Inversion eigenvalues of the minimal model at high-symmetry k-points. The band inversion happens at Γ, where there is an imbalance of even/odd eigenvalues in the irrep decomposition, just as in the DFT result in main text Table I.

$\boldsymbol{K}$	Γ	Y	T	Z
Inversion Eigenvalues	2+	1+ 1-	1+ 1-	1+ 1-

Minimal model details

In this section we provide full details on the minimal model. First, the only symmetries preserving the monolayer are inversion symmetry and $\{m_y|0\frac{1}{2}0\}$, resulting in a monolayer space group of $P2_1/m$ (#11). The Wyckoff positions for the monolayer model are

Figure 2: Surface properties of a glide-symmetric surface. a) Surface BZ highlighting glide symmetry protected paths. b) Schematic origin for the hourglass fermion dispersion in the surface spectrum.

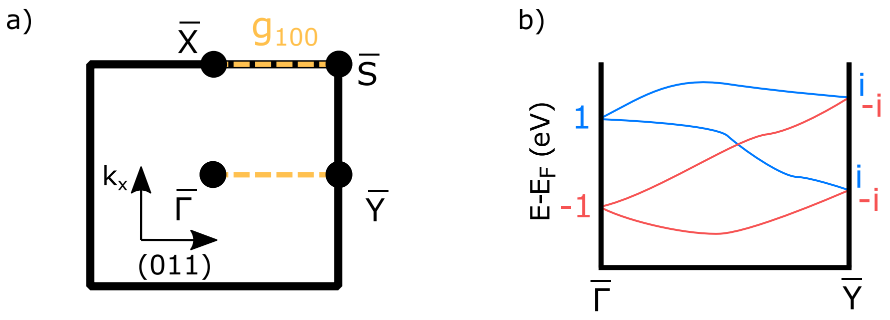

TABLE S 6: Numerical values of the interlayer coupling. All terms are written in units of meV.

	p_z-p_z (meV)	p_z-p_y (meV)	p_y-p_z (meV)	p_y-p_y (meV)
v	1.013	0.248	0.440	0.949

$q_0 = (0.499, 0.750, 0.217)$ and $q_2 = -q_0$. Each As atom has two neighbors, with hopping amplitudes denoted by t and r, ordered by increasing distance (see main text Fig. 3. Each bond involves three types of hopping: p_z-p_z, p_z-p_y, and p_y-p_y. The numerical values of these hopping amplitudes, obtained from Wannierization, are presented in Table 4.

Using the Mathematica package MagneticTB[53,54] we compute the inversion eigenvalues of the monolayer model at half filling and display them in Table 5, which give rise to a topological invariant $\mathbb{Z}_2 = 1$ as we explain in the main text. In order to couple the layers, we extract the hopping parameters from the wannierization. We only consider the nearest neighbors, which connects $q_0 - q_1$ and $q_2 - q_3$. They are related by symmetry, thus we only list the hoppings between $q_0 - q_1$ in Table 6.
